# Arabidopsis Transcriptomics Reveals the Role of Lipoxygenase2 (AtLOX2) in Wound-Induced Responses

**DOI:** 10.3390/ijms25115898

**Published:** 2024-05-28

**Authors:** Diljot Kaur, Andreas Schedl, Christine Lafleur, Julian Martinez Henao, Nicole M. van Dam, Jean Rivoal, Jacqueline C. Bede

**Affiliations:** 1 Department of Plant Science, McGill University, 21,111 rue Lakeshore, Ste-Anne-de-Bellevue, QC H9X 3V9, Canada; diljot.kaur@mail.mcgill.ca (D.K.); julian.martinezhenao@mail.mcgill.ca (J.M.H.); 2 Institut de Recherche en Biologie Végétale, Université de Montréal, 4101 rue Sherbrooke E., Montréal, QC H1X 2B2, Canada; jean.rivoal@umontreal.ca; 3 German Centre for Integrative Biodiversity Research (iDiv) Halle-Jena-Leipzig, Deutscher Platz 52, 04103 Leipzig, Germanyvandam@igzev.de (N.M.v.D.); 4 Institute of Biodiversity, Friedrich Schiller University Jena, 07743 Jena, Germany; 5 German Biomass Research Centre (DBFZ), Torgauer Straße 116, 04347 Leipzig, Germany; 6 Department of Animal Science, McGill University, 21,111 rue Lakeshore, Ste-Anne-de-Bellevue, QC H9X 3V9, Canada; christine.lafleur@mcgill.ca; 7 Leibniz Institute for Vegetable and Ornamental Crops (IGZ), Theodor-Echtermeyerweg-1, 14979 Großbeeren, Germany

**Keywords:** AtLOX2, jasmonate, 13*S*-lipoxygenase, transcriptome, wounding

## Abstract

In wounded *Arabidopsis thaliana* leaves, four 13*S*-lipoxygenases (AtLOX2, AtLOX3, AtLOX4, AtLOX6) act in a hierarchical manner to contribute to the jasmonate burst. This leads to defense responses with LOX2 playing an important role in plant resistance against caterpillar herb-ivory. In this study, we sought to characterize the impact of AtLOX2 on wound-induced phytohormonal and transcriptional responses to foliar mechanical damage using wildtype (WT) and *lox2* mutant plants. Compared with WT, the *lox2* mutant had higher constitutive levels of the phytohormone salicylic acid (SA) and enhanced expression of SA-responsive genes. This suggests that AtLOX2 may be involved in the biosynthesis of jasmonates that are involved in the antagonism of SA biosynthesis. As expected, the jasmonate burst in response to wounding was dampened in *lox2* plants. Generally, 1 h after wounding, genes linked to jasmonate biosynthesis, jasmonate signaling attenuation and abscisic acid-responsive genes, which are primarily involved in wound sealing and healing, were differentially regulated between WT and *lox2* mutants. Twelve h after wounding, WT plants showed stronger expression of genes associated with plant protection against insect herbivory. This study highlights the dynamic nature of jasmonate-responsive gene expression and the contribution of AtLOX2 to this pathway and plant resistance against insects.

## 1. Introduction

In addition to their roles in plant development, such as root elongation and pollen development [1,2], jasmonates are most recognized for their critical role in induced plant resistance to necrotrophic pathogens and chewing insect herbivores [3]. In *Arabidopsis thaliana* (arabidopsis), jasmonate biosynthesis is initiated from chloroplast membrane-derived galactolipids generating α-linolenic acid (18:3) upon wounding or recognition of pathogen or insect attack [1,4,5]. A stromal 13*S*-lipoxygenase (LOX) then catalyzes the oxygenation of α-linolenic acid at the 13C position, producing (13*S*)-hydroperoxyoctadecatrienoic acid (HPOA). Through sequential reactions catalyzed by allene oxide synthase (AOS) and allene oxide cyclase (AOC), HPOA is converted to the active phytohormone (9*S*,13*S*)-12-*oxo*-phytodienoic acid (OPDA), which is transported from the chloroplast by JASSY transporters [6,7,8]. Through the CTS/PXA1 transporters [9], OPDA is transported from the chloroplast to the peroxisome and converted to jasmonic acid (JA) through reduction followed by β-oxidation. JA is exported to the cytosol, possibly through the transporter AtJAT2 [10], where it is conjugated to isoleucine, a step catalyzed by jasmonate-resistant 1 (JAR1) [11], to form the bioactive (+)-7-*iso*-jasmonoyl-isoleucine (JA-Ile) [12]. The resultant JA-Ile enters the nucleus via AtJAT1 to bridge jasmonate-ZIM domain (JAZ) transcriptional repressors to the SCF^COI1^ complex that ubiquitinates the JAZ protein, targeting them for degradation by the 26*S*-proteosome [13,14,15]. Removal of JAZ proteins releases MYC transcription factors from the JAZ-NINJA-TOPLESS repressor complex, allowing MED25 to interact with the MYC transcription factors and jasmonate-responsive transcript expression to occur, leading to gene expression involved in plant defenses [16,17].

In arabidopsis, jasmonate signaling leads increased foliar levels of glucosinolates (GSLs), S- and N-rich specialized metabolites that contribute protection against insects and pathogens [18,19,20]. In the leaves, GSLs are spatially separated from their activating enzyme, myrosinase [21]. Herbivore damage disrupting foliar tissue allows contact between the myrosinases and GSLs, resulting in the hydrolysis of the thioester bond and releasing an unstable compound that undergoes rearrangement to produce toxic compounds such as isothiocyanates, thiocyanates and nitriles [18]. Though both aliphatic and indolic GSLs may negatively affect insect herbivores, in general, aliphatic GSLs have a stronger negative impact on generalist caterpillar herbivores while indolic GSLs negatively affect aphids [22,23,24,25,26].

Given the increase in jasmonates within seconds after wounding [27,28], jasmonate biosynthetic enzymes are thought to be constitutively present and regulated both transcriptionally and post-translationally in response to stress [29,30]. Indeed, an enzyme that catalyzes an early step in jasmonate biosynthesis, AtLOX2, is constitutively phosphorylated at Ser^600^ and dephosphorylated in wounded plants [31]. In vitro studies using phosphovariants confirmed that phosphorylation of AtLOX2 at Ser^600^ may affect enzyme activity with lower activity observed in the phosphomimics (Ser^600^-to-Asp, Ser^600^-to-Met) compared with the unphosphorylated WT (Ser^600^) or phosphonull variants (Ser^600^-to-Ala) [32]. This is likely due to the fact that Ser^600^ phosphorylation interferes with the positioning of the substrate on the enzyme active site.

13*S*-LOXs, such as AtLOX2, catalyze the addition of oxygen to the 13-position of a chloroplast membrane-derived polyunsaturated fatty acid, typically α-linolenic acid. In arabidopsis, there are four functional 13*S*-LOXs, AtLOX2, AtLOX3, AtLOX4 and AtLOX6, that contribute to the jasmonate burst. A hierarchy is noted where LOX2 and LOX6 activity results in jasmonate production that leads to the expression of *AtLOX2*, *AtLOX3* and *AtLOX4* [33]. Thus, AtLOX2 contributes to the early foliar jasmonate burst, as does AtLOX6, and is primarily associated with mesophyll and bundle sheath cells and important in local signaling [27,28,33]. In contrast, AtLOX6 is localized near the vasculature and likely plays an important role in long-range jasmonate signals. AtLOX3 and AtLOX4 are associated with the xylem and phloem, respectively, and are responsible for later (4 h after wounding) jasmonate biosynthesis, along with AtLOX2.

Even though these four functional AtLOXs contribute to jasmonate biosynthesis in response to plant stress, AtLOX2 is thought to be the most important for plant resistance against caterpillar herbivory [28,33]. Generalist caterpillars of the Egyptian cotton leafworm, *Spodoptera littoralis*, gained nearly twice the biomass when reared on the *lox2* mutant compared with WT or *lox3/4/6* plants [33]. As well, LOX2 activity results in singlet oxygen (^1^O_2_) production in wounded leaf tissue, which may affect transcriptional responses [34,35].

In this study, we seek to dissect the role of AtLOX2 in plant defense further by comparing wound-induced responses after foliar wounding of Col-0 and the loss-of-function *lox2* mutant. We combined bioinformatic analyses of transcriptomics data with phytohormone and GSL analyses focusing on phytohormone signaling and ^1^O_2_-responsive gene expression as well as GSL metabolism. Our results show that constitutive AtLOX2-associated jasmonate biosynthesis may be involved in antagonizing salicylic acid (SA) levels. In wounded arabidopsis, AtLOX2 exerts a high degree of control on the plants’ response to damage. In particular, the mechanically damage-associated jasmonate burst was lower in the *lox2* mutant, which is reflected in the reduced expression of late wound-induced genes.

## 2. Results

Arabidopsis has four functional 13*S*-LOXs involved in the biosynthesis of jasmonates in response to wounding or biotic stresses, such as insect herbivory or pathogens, making it complex to tease out the role of individual enzymes in plant resistance [36]. In wounded arabidopsis leaves, these proteins work in a hierarchical arrangement with AtLOX2, primarily contributing to plant resistance against caterpillar herbivory [33]. Using a *lox2* mutant [28], we conducted a time course transcriptomic experiment to understand the role of AtLOX2 in the plant response to wounding, particularly in connection to plant protection.

### 2.1. Transcriptome Profiling and Mapping onto the Arabidopsis Genome

After processing to remove the adaptor and poor-quality reads, RNA-Seq produced an average of 39.7 million 100 bp reads per sample, all with a Pfred score at or above 20. The high-quality reads were aligned with the arabidopsis TAIR10 genome with an average mapping efficiency of 97.8% uniquely mapped reads (Supplemental Table S4).

### 2.2. Constitutive Phytohormone and Gene Expression Levels

**Figure 1:**
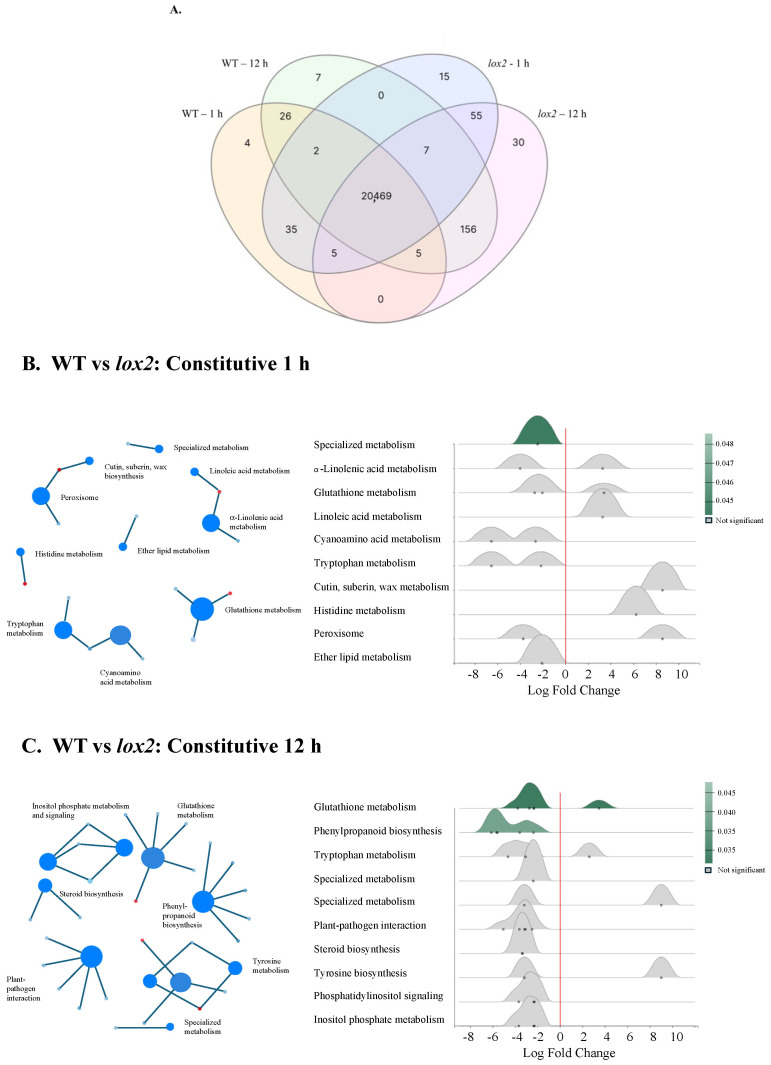
Foliar constitutive gene expression. (**A**): Venn diagram of constitutive genes differentially expressed between wildtype (WT) and *lox2 Arabidopsis thaliana* plants at two time points (1 and 12 h). Differentially expressed genes between the different treatments and times were determined by DESeq2 (*p*-value (padj) ≤ 0.05 and log2 fold change ≥2 or ≤−2). (**B**): Gene set enrichment analysis (GSEA) and ridgeline plot highlighting constitutive metabolic pathways differentially expressed between WT and *lox2* plants at 1 h. (**C**): GSEA and ridgeline plot highlighting constitutive metabolic pathways differentially expressed between WT and *lox2* plants at 12 h. The ridgeline plot visualizes the distribution of differential enrichment categories identified by GSEA. Significantly differentially expressed pathways in ridgeline plots are indicated in green.

**Figure 2:**
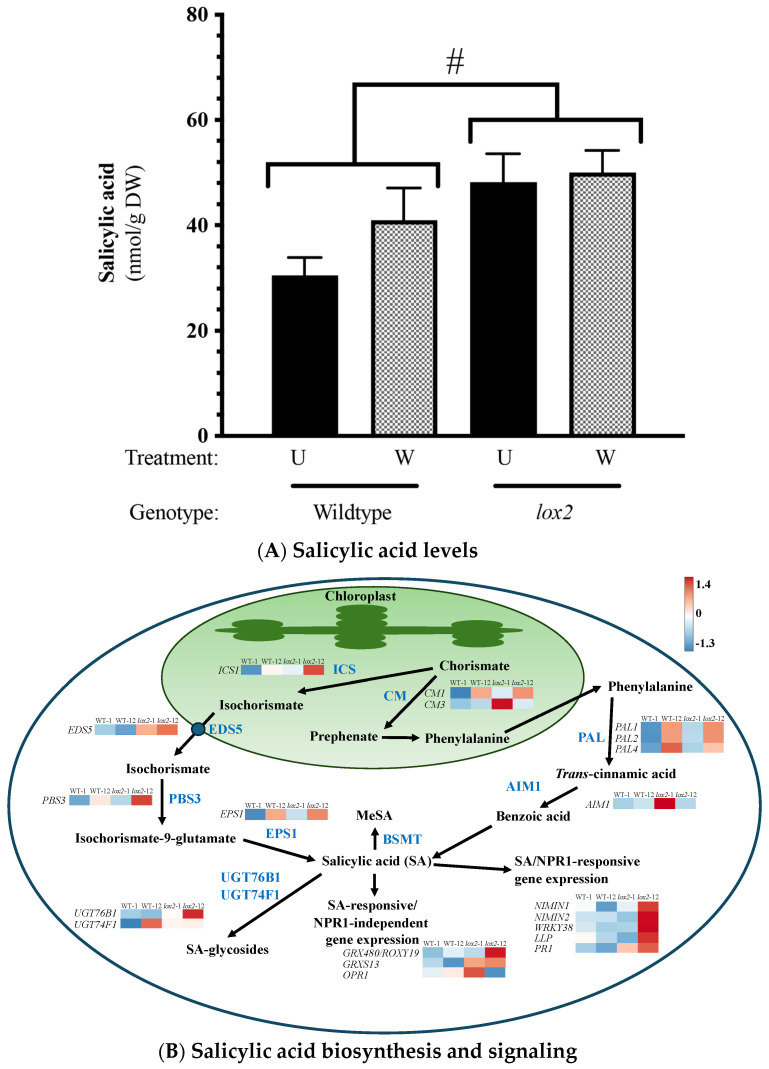
Foliar salicylic acid (SA) levels and SA-related gene expression. Four-week-old *Arabidopsis thaliana* wildtype (WT) or *lox2* plants were left undamaged (U) or wounded (W) with a hole punch on each fully expanded rosette leaf and harvested 1 or 12 h post-damage. (**A**): Foliar salicylic acid (SA) levels. (**B**): Constitutive expression of genes involved in SA biosynthesis and SA-responsive gene expression. Phytohormone levels are represented by the mean ± SE. Differences in phytohormone levels were determined by two-factor analysis of variance (2-factor ANOVA) (factors: genotype, treatment) followed by Tukey HSD (Supplemental Table S2). A hashtag (#) indicates genotype differences. Heatmaps visualize constitutive gene expression (wildtype—1 h (WT-1), wildtype—12 h (WT-12), *lox2*—1 h (*lox2*-1), *lox2*—12 h (*lox2*-12)). Genes: *ICS1/SID2/EDS16* (*At1g74710*), *EDS5* (*At4g39030*), *PBS3* (*At5g13320*), *EPS1* (*At5g67160*), *CM1* (*At3g29200*), *CM3* (*At1g69370*), *PAL1* (*At2g37040*), *PAL2* (*At3g53260*), *PAL4* (*At3g10340*), *AIM1* (*At4g29010*), *NIMIN1* (*At1g02450*), *NIMIN2* (*At3g25882*), *WRKY38* (*At5g22570*), *LLP* (*At5g03350*), *PR1* (*At2g14610*), *GRX480/ROXY19* (*At1g28480*), *GRXS13* (*At1g03850*), *OPR1* (*At1g76680*), *UGT74F1* (*At2g43840*), *UGT76B1* (*At3g11340*), *BSMT1* (*At3g11480)*.

**Figure 3:**
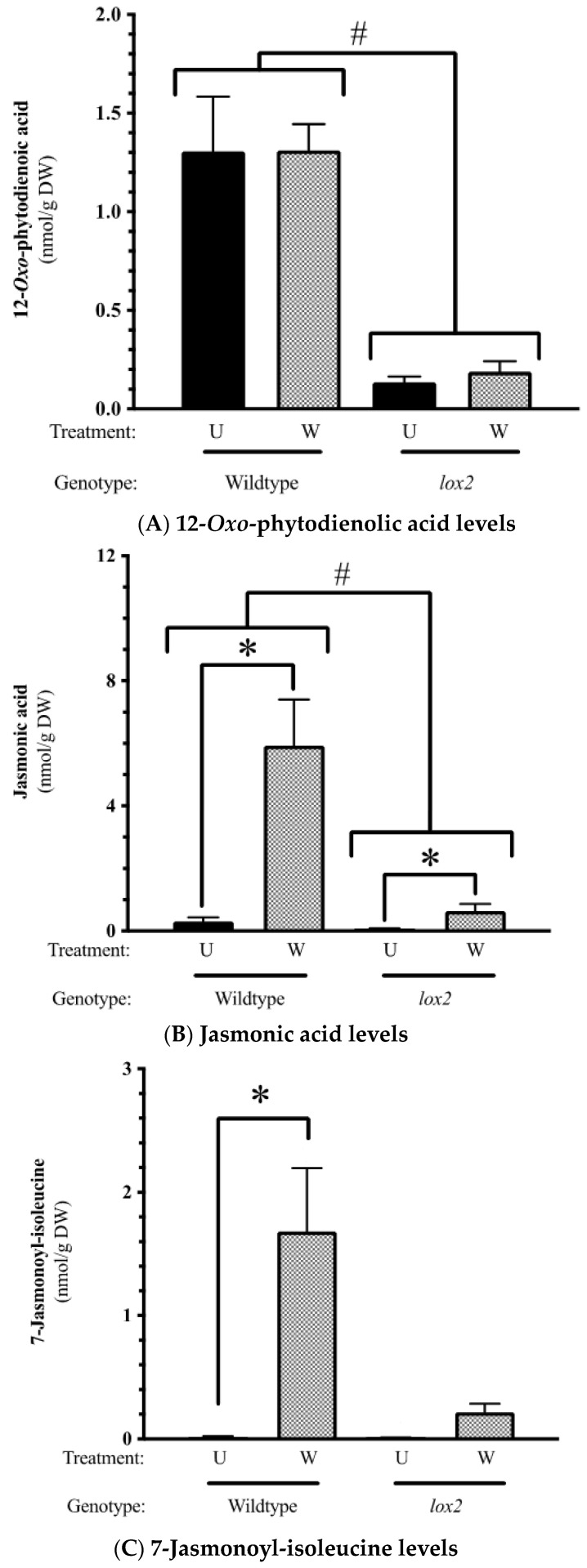

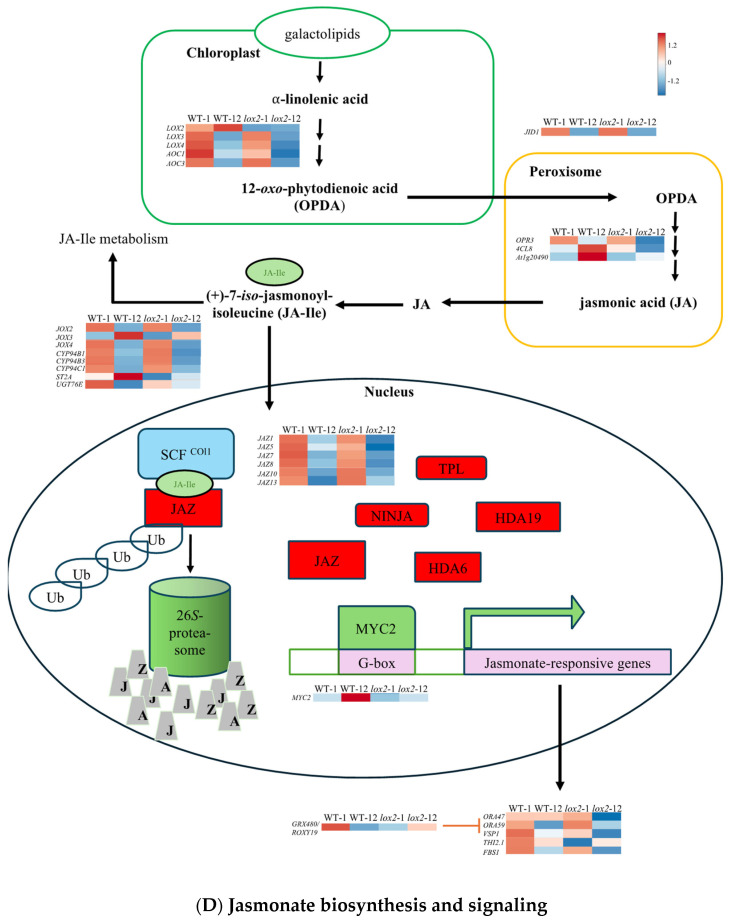
Foliar jasmonate levels and jasmonate-related gene expression. Four-week-old *Arabidopsis thaliana* wildtype (WT) or *lox2* plants were left undamaged (U) or wounded (W) with a hole punch on each fully expanded rosette leaf and harvested 1 or 12 h post-damage. Jasmonate levels 1 h after wounding: (**A**): 12-*oxo*-phytodienoic acid (OPDA), (**B**): jasmonic acid and (**C**): 7-jasmonyl-isoleucine (JA-Ile). (**D**): Foliar wound-induced jasmonate-related gene expression (1 and 12 h). Phytohormone levels are represented by the mean ± SE. Differences in phytohormone levels were determined by two-factor analysis of variance (2-factor ANOVA) (Factors: genotype, treatment) followed by Tukey HSD (Supplemental Table S2). An asterisk (*) indicates wound-induced phytohormone levels and a hashtag (#) represents genotype differences. Wound-induced genes were determined by DESeq2 (*p*-value (padj) ≤ 0.05 and log2 fold change ≥2 or ≤−2). Heatmaps visualize wound-induced gene expression (wildtype—1 h (WT-1), wildtype—12 h (WT-12), *lox2*—1 h (*lox2*-1), *lox2*—12 h (*lox2*-12)). Genes: *LOX2 (AT3g45140)*, *LOX3 (At1g17420)*, *LOX4 (At1g72520)*, *AOC1* (*At3g25760*), *AOC3* (*At3g25780*), *OPR3* (*At2g06050*), *OPCL1* (*At1g20510*), *At1g20490*, *FBS1* (*At1g61340*), *MYC2* (*At1g32640*), *JAZ1* (*At1g19180*), *JAZ5* (*At1g17380*), *JAZ7* (*At2g34600*), *JAZ8* (*At1g30135*), *JAZ10* (*At5g13220*), *JAZ13* (*At3g22275*), *JOX2* (*At5g05600*), *JOX3* (*At3g55970*), *JOX4* (*At2638240*), *CYP94B1* (*At5g63450*), *CYP94B3* (*At3g48520*), *CYP94C1* (*At2g27690*), *ST2A* (*At5g07010*), *JID1* (*At1g06620*), *ORA47* (*At1g74930*), *ORA59* (*At1g6160*), *VSP1* (*At5g24780*), *THI2.1* (*At1g72260*), *GRX480/ROXY19* (*At1g28480*).

Constitutively, gene expression was highly conserved between the two genotypes (~20,469 genes) (Figure 1A); less than 1% were differentially expressed between WT and *lox2* at any time point. In WT plants compared with *lox2* mutants, there was an increase in specialized metabolism, glutathione metabolism and phenylpropanoid biosynthesis (Figure 1B,C). However, some pathogen resistance-related genes had higher expression levels in *lox2* plants compared with WT plants, which may reflect the increased SA levels in these plants (Figure 2). These transcripts included genes that encode enzymes in SA biosynthesis (i.e., AtICS1, AtCM3, AtAIM1 [37]) and SA-responsive genes (i.e., *AtPR1*, *AtNIMIN1*, *AtNIMIN2*, *AtGRXS13* [38]). In contrast, constitutive levels of the jasmonate phytohormone OPDA and JA were higher in WT plants compared with the *lox2* mutant (Figure 3A,B).

### 2.3. Arabidopsis Responses to Foliar Damage

After wounding, the levels of JA and JA-Ile rose in damaged rosette leaves in both or only WT genotypes, respectively (Figure 3B,C). As expected, levels of these phytohormones were ~7.5 times higher in WT than the *lox2* mutant. Jasmonate signaling is dynamic with the early expression of genes encoding biosynthetic enzymes and positive regulators (Figure 3D) [39,40,41]. In addition, the jasmonate burst is tempered either by catabolizing jasmonates to a less active form or by the activity of JAZ proteins that bind to and repress MYC2/3/4 transcription factors [42,43].

**Figure 4:**
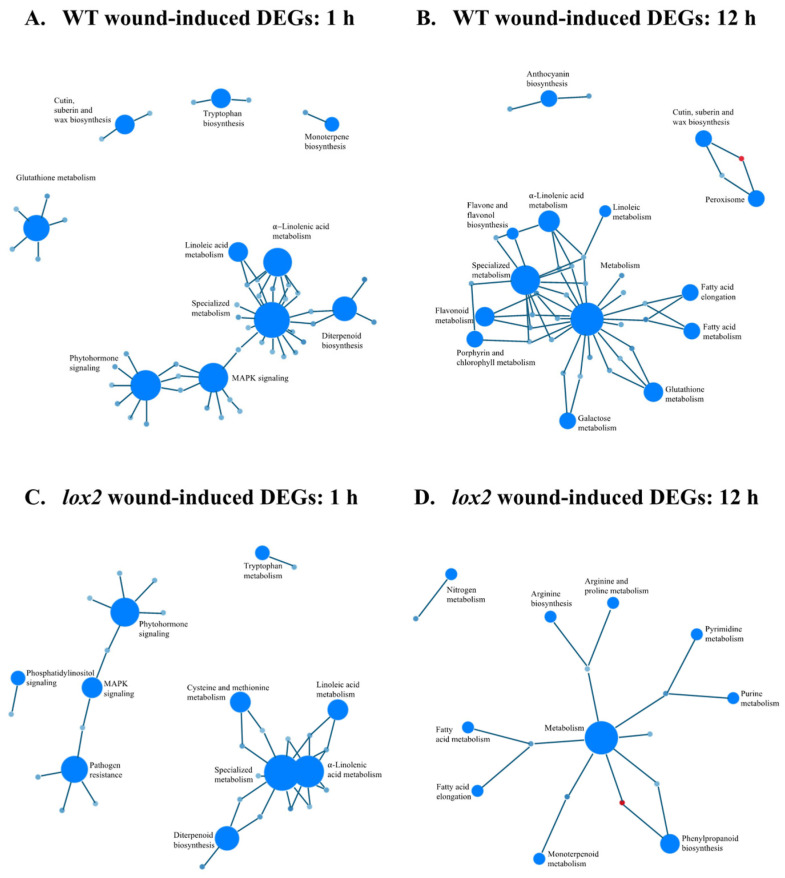

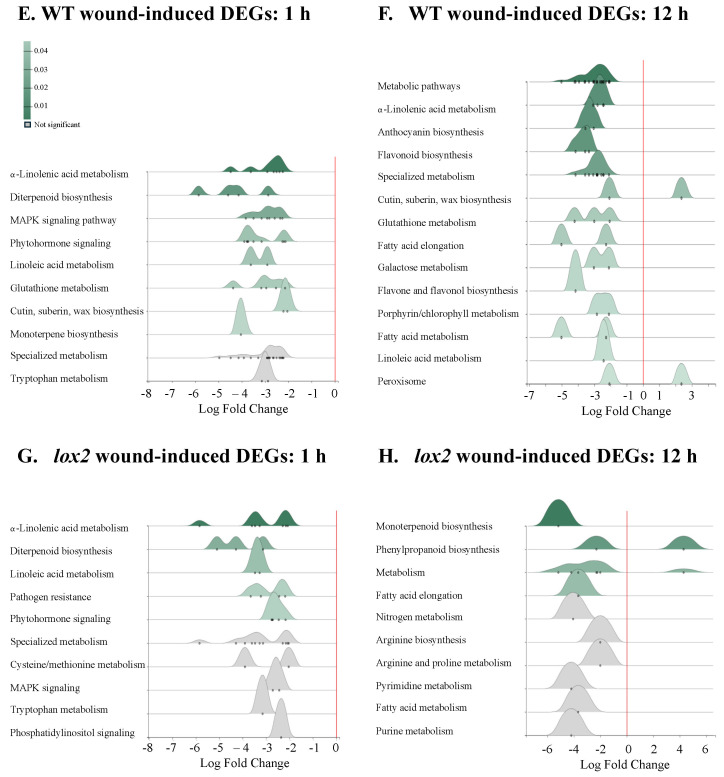
Wound-induced foliar gene expression. Four-week-old *Arabidopsis thaliana* wildtype (WT) or *lox2* plants were wounded with a hole punch on each fully expanded rosette leaf and harvested at 1 and 12 h. Gene set enrichment analyses (GSEA) and ridgeline plots of WT (1 h) (**A**,**E**), WT (12 h) (**B**,**F**), *lox2* (1 h) (**C**,**G**) and *lox2* (12 h) (**D**,**H**). Wound-induced differentially expressed genes (DEGs) were determined by DESeq2 (*p*-value (padj) ≤ 0.05 and log2 fold change ≥2 or ≤−2). The ridgeline plot visualizes the distribution of differential enrichment categories identified by GSEA. Significantly differentially expressed pathways in ridgeline plots are indicated in green.

In WT plants, 324 genes were differentially expressed in response to wounding, with 252, 58 and 14 expressed early, late or at both time points, respectively. At 1 h after wounding, a strong increase in the expression of transcripts that encode proteins involved in α-linolenic and linoleic acid biosynthesis, phytohormone and MAPK signaling, terpenoid biosynthesis and wound healing (cutin, suberin and wax biosynthesis) as well as redox metabolism (glutathione metabolism) was observed (Figure 4A,B,E,F). Though most of the processes observed 1 h post-damage were also observed later, there was also an increase in primary metabolic pathways 12 h after wounding. In contrast, though the same general pathways were upregulated in the *lox2* mutant (Figure 4C,D), fewer genes were strongly expressed (Figure 4G,H).

**Figure 5:**
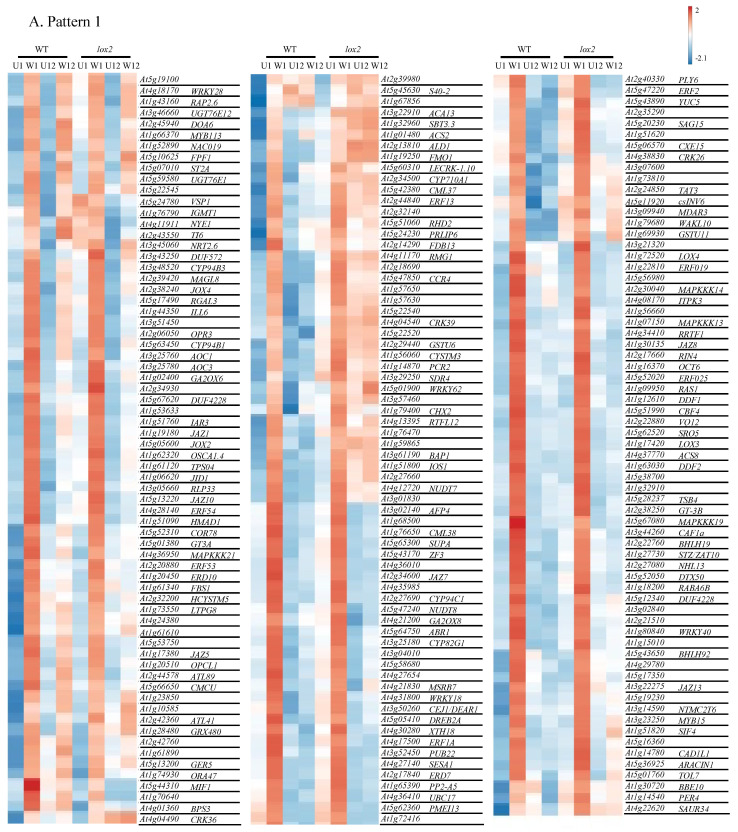

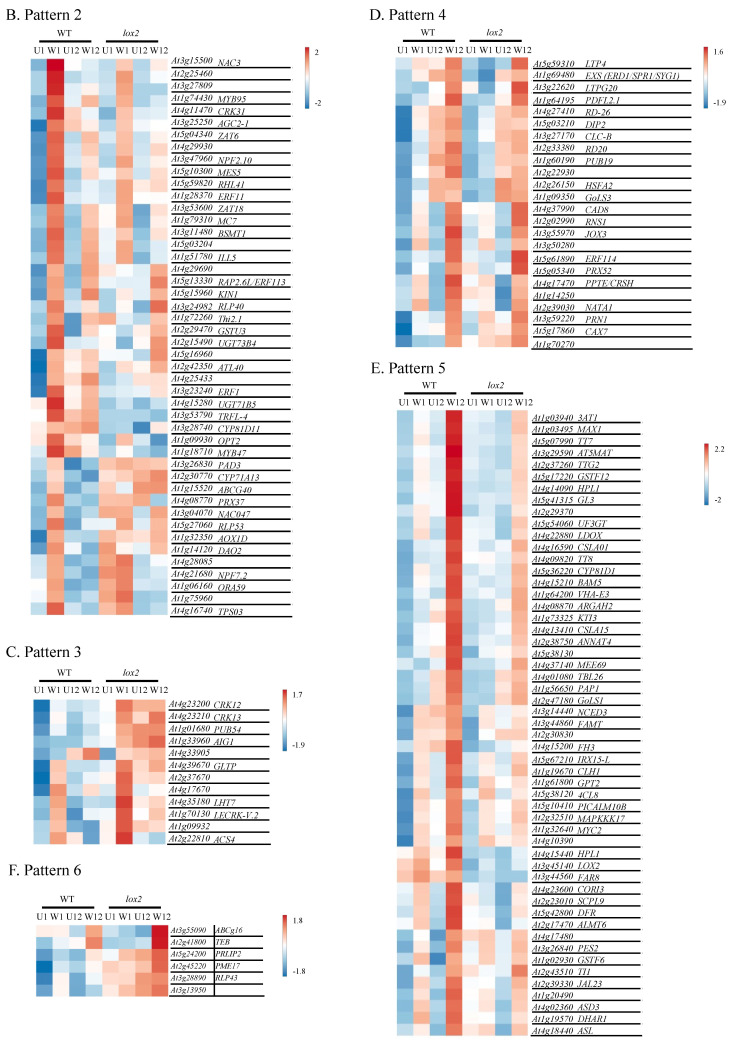
Wound-induced foliar gene expression: Patterns. Four-week-old *Arabidopsis thaliana* wildtype (WT) or *lox2* plants were wounded with a hole punch on each fully expanded rosette leaf and harvested at 1 and 12 h. Wound-induced genes fell into six general expression patterns visualized by heatmaps. Early gene expression (peak at 1 h): (**A**) general-both genotypes, (**B**) WT and (**C**) *lox2*. Later gene expression (peak 12 h): (**D**) general-both genotypes, (**E**) WT and (**F**) *lox2*. Wound-induced genes were determined by DESeq2 (*p*-value (padj) ≤ 0.05 and log2 fold change ≥2 or ≤−2) (Supplemental Table S5). Heatmaps visualize gene expression (undamaged-U, wound-W, 1 h-1, 12 h-12).

In general, WT and *lox2* plants showed similar wound-induced responses (Figure 5A,D). One hour post-damage, out of the 249 wound-induced genes observed in WT plants, 81% were also induced in damaged *lox2* (Figure 5A,B). A stronger difference in wound-induced gene expression between WT and *lox2* plants was observed 12 h after wounding (Figure 5D,E). At this time point, only 76 wound-induced genes were identified in WT and shared 31% gene expression with damaged *lox2*.

Based on this, wound-induced gene expression patterns were divided into six distinct groups as follows: Pattern 1—general early (1 h) wound-induced gene expression (Figure 5A), Pattern 2—early (1 h) wound-induced gene expression with higher expression in WT plants (Figure 5B), Pattern 3—early (1 h) wound-induced gene expression with higher expression in *lox2* plants (Figure 5C), Pattern 4—general late (12 h) wound-induced gene expression (Figure 5D), Pattern 5—late (12 h) wound-induced gene expression with higher expression in WT plants (Figure 5E) and Pattern 6—late (12 h) wound-induced gene expression with higher expression in *lox2* plants (Figure 5F).

### 2.4. Pattern 1: General Early Wound-Induced Gene Expression

In this group, wound-induced gene expression is higher at 1 h and typically returned to near-basal levels at 12 h in both genotypes (Figure 5A, Supplemental Table S5A). One hour post-damage, the expression of genes encoding proteins involved in jasmonate biosynthesis and signaling were strongly upregulated in both genotypes (i.e., AtLOX3, AtLOX4, AtLOX6, AtAOC1, AtAOC3, AtOPR3, AtOPCL1 and AtORA47 [39,40,41]) (Figure 3D). In addition, genes encoding proteins involved in jasmonate anabolism (i.e., AtJOX2, AtJOX4, AtCYP94B1, AtCYP94B3, AtILL6 and AtJID1 [44,45,46,47,48]) and JAZ proteins (AtJAZ1, AtJAZ2, AtJAZ5, AtJAZ7, AtJAZ8, AtJAZ10, AtJAZ13 [42,43]) were also induced.

In line with this, jasmonate-responsive genes were among the early responding genes including those that encode enzymes in volatile biosynthesis (i.e., AtTPS04/GES, AtCYP82G1 [49,50]), enzymes involved in sealing damaged tissue (i.e., AtPP2-A5 [51]), transcription factors (i.e., AtRRTF1, AtWRKY40, AtRAP2.6 [52,53,54,55,56]) and proteins involved in plant defense against pathogens or insects (i.e., AtTI6, AtMAPKKK21 [57,58]).

Gene expression associated with other phytohormones and their signaling pathways that may be involved in crosstalk were also observed. Wound-induced expression of *AtGA2OX6* and *AtGA2OX8*, encoding enzymes that oxidize gibberellins lowering their availability [59], as well as the negative growth regulator DELLA protein *AtRGAL3* [60,61], may reflect the shift from growth to defense [62]. Even though abscisic acid (ABA) levels were not affected by wounding (Supplemental Figure S1), a number of ABA-related genes were induced 1 h after wounding. The expression of the ABA receptor *AtPYL6* and ABA-responsive genes (i.e., *AtRAS1*, *AtERD7*, *AtOSCA1.4*, *AtERD10*, *AtCOR78* [63,64,65,66,67]) were upregulated early after foliar damage. ABA-related responses help minimize water loss from damaged leaves as well as enhance plant defense responses [68,69,70]. In response to wounding, synergistic defensive responses between jasmonates and ethylene are often observed [71]; genes encoding ethylene biosynthetic enzymes (i.e., AtCSP2 and AtACS8) and ethylene responses (i.e., AtERF2 and AtRAP2.6) were noted in early wound-induced responses.

LOX2 has been implicated in wound-associated, chloroplastic generation of ^1^O_2_ [34,35]. Comparing transcript expression of 66 ^1^O_2_-responsive genes in our damaged foliar tissues 1 h post-wounding [72,73], a genotype-difference in wound-induced ^1^O_2_-responsive transcript expression was not observed (Supplemental Figure S2).

### 2.5. Pattern 2: Early Wound-Induced Gene Expression in WT Plants

In general, genes in this group were often wound-induced in both WT and *lox2* but showed earlier, higher expression levels in WT plants (Figure 5B, Supplemental Table S5B). These early damage-induced genes include transcriptional regulators of jasmonate responses (i.e., *AtORA59* [74]) as well as jasmonate- and wound/insect-responsive genes (i.e., *AtTHI2.1*, *AtTPS03*, *AtCYP81D11* [75,76,77,78]).

### 2.6. Pattern 3: Early Wound-Induced Gene Expression in lox2 Plants

Likely reflecting elevated SA levels (Figure 2, Supplemental Table S5C), SA-responsive genes (*AtAIG1*, *AtCRK13* [79,80]) were expressed at higher levels in wounded *lox2* mutants compared with WT plants (Figure 5C).

### 2.7. Pattern 4: General Late Wound-Induced Gene Expression

**Figure 6:**
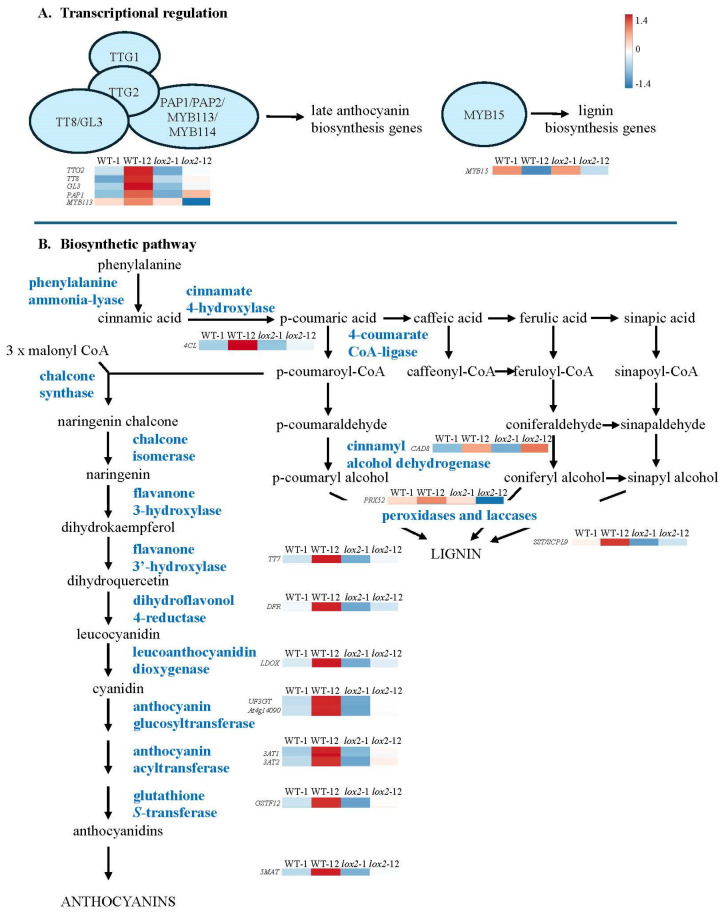
Wound-induced genes involved in polyphenol biosynthesis. Four-week-old *Arabidopsis thaliana* wildtype (WT) or *lox2* plants were wounded with a hole punch on each fully expanded rosette leaf and harvested at 1 or 12 h. (**A**) Transcriptional regulators and (**B**) biosynthetic genes. Wound-induced genes were determined by DESeq2 (*p*-value (padj) ≤ 0.05 and log2 fold change ≥2 or ≤−2). Heatmaps visualize wound-induced gene expression (wildtype—1 h (WT-1), wildtype—12 h (WT-12), *lox2*—1 h (*lox2*-1), *lox2*—12 h (*lox2*-12)). Genes: *TTG2* (*At2g37260*), *TT8* (*At4g09820*), *GL3* (*At5g41315*), *PAP1* (*At1g56650*), *MYB113* (*At1g66379*), *4CL* (*At1g20490*), *TT7* (*At5g07990*), *DFR* (*At5g42800*), *LDOX* (*At4g22880*), *UF3GT* (*At5g54060*), *At4g14090*, *3AT1* (*At1g03840*), *3AT2* (*At1g03495*), *GSTF12* (*At5g17220*), *5MAT* (*At3g29590*), *MYB15* (*At3g23250*), *CAD8* (*At4g37990*), *PRX52* (*At5g05340*), *SST/SCPL9 *(*At2g23010*).

Pattern 4 shows wound-induced genes in both genotypes that were more highly expressed 12 h after mechanical damage (Figure 5D, Supplemental Table S5D). Late wound-induced genes in both plant genotypes include *AtPDFL2.1*, *AtNATA1*, *AtRD20*, *AtPRN1*, *AtPPTE/AtCRSH* and *AtRNS1*. Genes encoding enzymes in lignin biosynthesis (i.e., AtCAD8, AtPRX52) may also contribute to wound-induced lignin deposition (Figure 6) [81,82].

### 2.8. Pattern 5: Late Wound-Induced Gene Expression in WT Plants

Most late-induced genes showed higher expression in WT plants compared with *lox2* plants 12 h post-damage (Figure 5E, Supplemental Table S5E). This likely reflects the expression of genes encoding enzymes in jasmonate biosynthesis (AtLOX2, At4CL8) and signaling (AtMYC2) that were more highly expressed in wounded WT compared with *lox2* plants [4]. Jasmonate-responsive genes involved in plant resistance to insect herbivory, such as *AtHPL/AtCYP74B2*, which encodes an enzyme involved in volatile biosynthesis [83], *AtCLH1*, *AtKTI3*, *AtTI1*, *AtARGAH2* and *AtMAPKKK17*, reflect this pattern [58,84,85,86]. Genes involved in antioxidant pathways are upregulated in wounded WT plants, including those that encode proteins involved in the ascorbate/glutathione cycle (i.e., AtDHAR1 [87]) and anthocyanin biosynthesis (i.e., AtPAP1, AtTT8, AtGL3, AtTTG2, At4CL, AtTT7, AtDFR, AtLDOX, AtUF3GT, At1g14090, At3AT1, At3AT2, AtGSTF12, At5MAT [88,89,90]) (Figure 6). Of note, AtTTG1 and AtGL3 also regulate trichome development [91].

### 2.9. Pattern 6: Late Wound-Induced Gene Expression in lox2 Plants

Only a few genes showed *lox2*-specific late wound-induced expression (Figure 5F, Supplemental Table S5F). Expression of the SA-responsive gene *AtPRLIP2* was observed in wounded *lox2* mutants [92,93].

### 2.10. Glucosinolates

**Figure 7:**
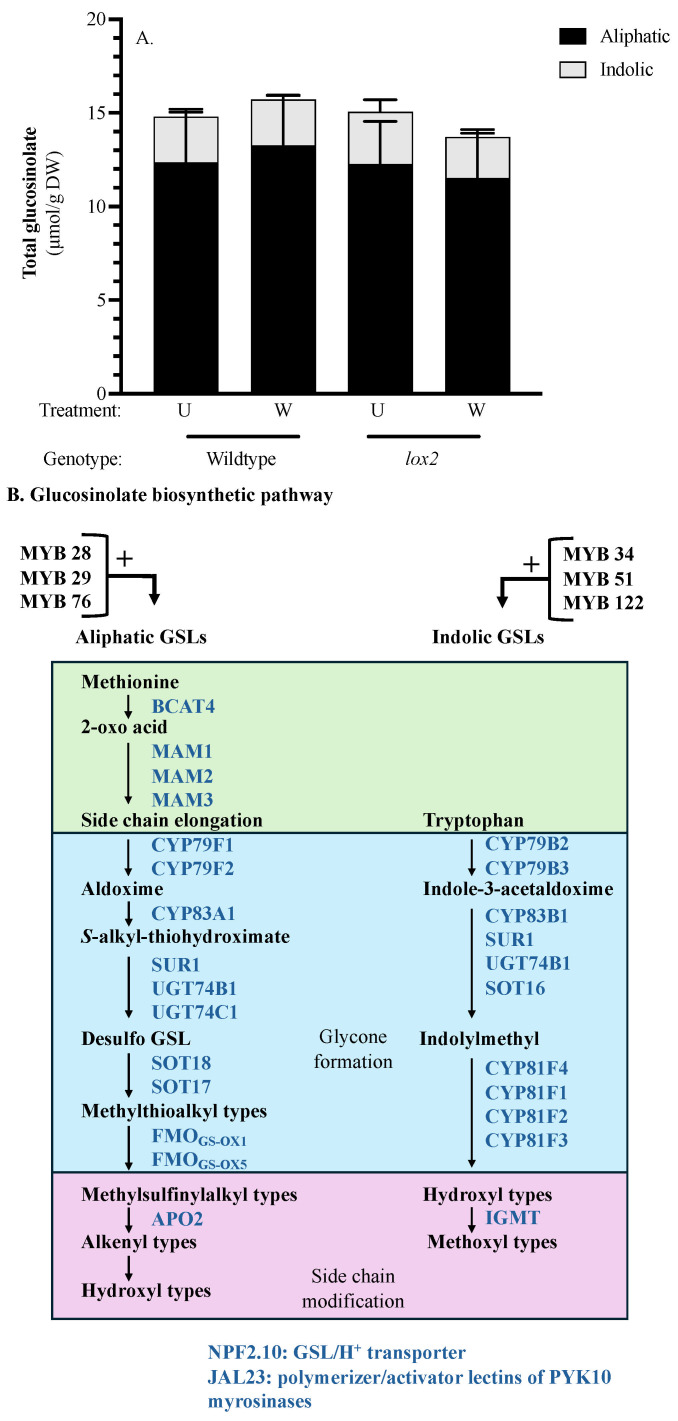

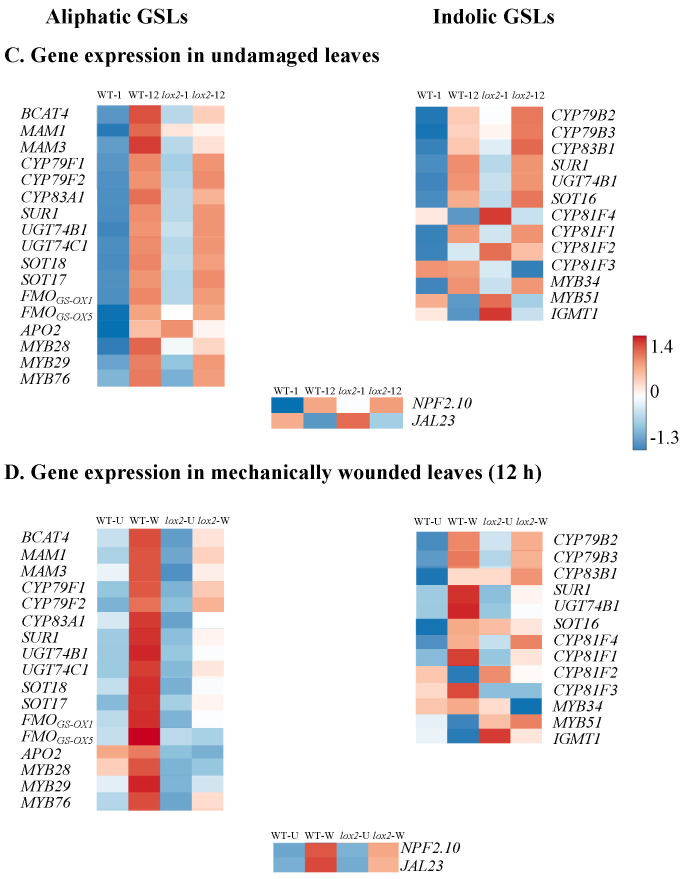
Foliar glucosinolate (GSL) levels and expression of GSL biosynthesis genes. Four-week-old *Arabidopsis thaliana* wildtype (WT) or *lox2* plants were wounded with a hole punch on each fully expanded rosette leaf and harvested at 1 and 12 h. (**A**) Foliar aliphatic and indolic GSL levels in undamaged (U) and wounded (W) WT and *lox2* plants taken 12 h after mechanical damage. (**B**) GSL pathway illustrating transcriptional activators, biosynthetic enzymes and other GSL-related proteins. Gene expression in (**C**) undamaged and (**D**) mechanically damaged arabidopsis rosettes. Glucosinolate levels are represented by the mean ± SE. Differences in GSL levels were determined by a two-factor analysis of variance (2-factor ANOVA) (factors: genotype (WT or *lox2*), treatment) followed by Tukey HSD (Supplemental Table S3). Heatmaps visualize gene expression (for C: wildtype—1 h (WT-1), wildtype—12 h (WT-12), *lox2*—1 h (*lox2*-1), *lox2*—12 h (*lox2*-12); for D: wildtype undamaged—WT-U, wildtype wounded—WT-W, *lox2* undamaged—*lox2*-U, lox2 wounded—*lox2*-W)). Genes: *BCAT4* (*At3g19710*), *MAM1* (*At5g23010*), *MAM3* (*At5g23020*), *CYP79F1* (*At1g16410*), *CYP79F2* (*At1g16400*), *CYP83A1* (*At4g13770*), *SUR1* (*At2g20610*), *UGT74B1* (*At1g24100*), *UGT14C1* (*At2g31790*), *SOT18* (*At1g74090*), *SOT17* (*At1g18590*), *FMO_GS-OX1_* (*At1g65860*), *FMO_GS-OX5_* (*At1g12140*), *APO2* (*At5g57930*), *MYB28* (*At5g61420*), *MYB29* (*At5g07690*), *MYB76* (*At5g07700*), *NPF2.10* (*At3g47960*), *JAL23* (*At2g39330*), *CYP79B2* (*At4g39950*), *CYP79B3* (*At2g22330*), *CYP83B1* (*At4g31500*), *UGT74B1* (*At1g24100*), *SOT16* (*At1g74100*), *CYP81F4* (*At4g37410*), *CYP81F1* (*At4g37430*), *CYP81F2* (*At5g57220*), *CYP81F3* (*At4g37400*), *IGMT1* (*At1g21100*), *MYB34* (*At5g60890*), *MYB51* (*At1g18570*).

Constitutively or 12 h post-wounding, the foliar GSL profile did not differ between the two genotypes, reflecting the gene expression profile of GSL biosynthetic enzymes (Figure 7A). However, genes encoding GSL transcriptional regulators and biosynthetic enzymes show a strong diurnal cycle with stronger expression in the light phase, particularly for genes involved in aliphatic GSL biosynthesis (Figure 7B). Genes encoding indole GSL methyltransferase1 (AtIGMT1), the GSL transporter AtNPF2.10 and lectin JAL23 (a polymerization factor and putative activator of the myrosinase PYK10 [94]) showed strong wound-induction in WT plants.

## 3. Discussion

Our results highlight the transcriptional dynamics of wound-induced jasmonate biosynthesis and the importance of AtLOX2 in sustained jasmonate signaling leading to plant resistance against insect herbivores.

### 3.1. The Dynamic Jasmonate Burst

Early wound-induced genes in both genotypes include enzymes in the jasmonate biosynthetic pathway (i.e., AtLOX3, AtLOX4, AtAOC1, AtAOC3, AtOPR3, AtOPCL1 [4]) and transcriptional regulators (i.e AtORA47) (Pattern 1, Figure 5A). ORA47 coordinates the expression of genes that encode jasmonate and ABA biosynthetic enzymes as well as in the general jasmonate-responsive stress network [41,95,96]. The expression of later genes that encode jasmonate biosynthetic enzymes (i.e., AtLOX2, At4CL8, At1g20490 (putative) [4,97]) were generally higher in WT compared with *lox2* plants (Pattern 5, Figure 5E). This was reflected in the expression of jasmonate-responsive gene expression, particularly in late wound-induced genes (Figure 5).

The wound-induced jasmonate burst is dynamic, and the initial strong jasmonate wave is dampened over time. Indeed, early wound-induced transcriptional expression included genes encoding proteins that attenuate the jasmonate-mediated signaling response, in particular, enzymes that metabolize jasmonates to their inactive form (i.e., AtST2A, AtJOX2, AtJOX4, AtCYP94B1, AtCYP94B3, AtILL6, AtJID1) and JAZ proteins (i.e., AtJAZ1, AtJAZ5, AtJAZ7, AtJAZ8, AtJAZ10, AtJAZ13) (Pattern 1, Figure 5A). JAZ proteins are negative regulators that bind to jasmonate signaling MYC2, MYC3 and MYC4 transcription factors [42,43]. Early wound-induced *JAZ* transcripts encode AtJAZ1, AtAtJAZ5, AtJAZ8, AtJAZ10 and AtJAZ13, which interact with AtMYC2 or AtMYC3, as well as AtJAZ7, which interacts with AtMYC2 [98,99,100].

In addition, early wound-induced genes include a number of enzymes that convert jasmonates into an potentially inactive forms (Pattern 1, Figure 5A). 2-*oxo*-glutarate-dependent dioxygenase genes *jasmonate-induced oxidase2* (*AtJOX2*) and *AtJOX4* as well as the aminohydrolase *AtILL6* are expressed early after wounding, while *AtJOX3* is expressed later (Figure 3); these genes encode enzymes that hydroxylate JA to a biologically inactive form [45,46,47]. Subsequently, the hydroxylated JA can be further metabolized by sulfotransferase 2A (ST2A) to the sulfated form [101]. Furthermore, the early wound-induced cytochrome P_450_ genes encoding AtCYP94B1, AtCYP94C1 and AtCYP94B3 potentially work in sequence to catalyze the formation of 12-OH-JA-Ile and 12-COOH-JA-Ile, respectively [44,102,103,104]. JID1 is a cytosolic enzyme thought to be involved in OPDA anabolism [48].

Often working synergistically, ethylene and jasmonate signaling leads to increased plant resistance [105]. Early genes encoding enzymes in ethylene biosynthesis, AtACS2, AtACS8 and AtACS4, as well as the transcription factors AtRAP2.6, AtERF2 and AtERF114, were wound-induced in WT or both genotypes 1 h post-damage (Figure 5A,C).

In comparison, SA and jasmonates often have an antagonistic relationship that acts to shape the plant defense response [106]. In general, the *lox2* mutant expressed higher constitutive expression of genes involved in SA biosynthesis (i.e., *AtICS*, *AtEDS5*, *AtPBS3*, *AtCM3*, *AtAIMI* [37,107]) that translated into higher SA levels and SA-responsive gene expression (i.e., *AtGRX480/AtROXY19*, *AtCRXS13*, *AtOPR1*, *AtNIMIN1*, *AtNIMIN2*, *AtWRKY38*, *AtLLP*, *AtPR1*) (Figure 2). Of these, *AtNIMIN1*, *AtNIMIN2* and *AtWRKY38*, which encode negative regulators of basal immunity [108,109,110], were expressed in the light (9 am), which may reflect the diurnal rhythm of SA, which is lower in the morning [111]. In wounded plants, SA-responsive *AtCRK13, AtAIG1* and *AtPRLIP2* were more highly expressed in *lox2* compared with WT plants (Pattern 3, Figure 5C; Pattern 6, Figure 5F) [79,80,92,93].

### 3.2. Plant Resistance against Insect Herbivory

#### 3.2.1. Response to Egg Deposition

In some species, plants respond to insect eggs by eliciting an SA-dependent “hypersensitive-like response” that results in necrotrophic tissue forming under the eggs, often leading to egg desiccation and plant resistance [112]. Treatment of leaves by spider mite egg extract resulted in the induction of genes encoding enzymes in the jasmonate biosynthesis and signaling pathway (i.e., AtAOC1, AtAOC3, AtLOX2, AtMYC2, AtJAZ9, AtST2A, AtJOX2, AtJOX3, AtJOX4, AtILL6, AtCYP82G1, AtMDHAR, AtFAMT, AtNATA1, AtZAT10/STZ, AtBSMT1, RNS1 At3g23350 and AtCORI3), which were also seen in our study (Figure 3D and Figure 5) [113].

#### 3.2.2. Signal Transduction

In this and other studies, AtMAPKKK17, induced late in response to wounding in WT plants (Pattern 5, Figure 5E), activates the MKK3-MPK1/2/7 module [114,115]. In plants challenged with the two-spotted spider mite, *Tetranchus urtica*, plants with higher constitutive *AtMAPKKK17* expression had higher resistance against mite herbivory, exhibiting less leaf damage with mites having lower fecundity [58]. *AtMAPKKK21* is expressed early after wounding in both genotypes (Pattern 1, Figure 5A). In contrast to MAPKKK17, this kinase was found to be a negative regulator of plant resistance to mites [58].

#### 3.2.3. Physical Defense

Foliar trichomes create a mechanical defense against insect herbivores and, as well, may be a site for the production of chemical defenses [116]. Even though arabidopsis trichomes are non-glandular, volatile organic compounds as well as aliphatic and indolic GSLs may be biosynthesized in these cells [21,50,117]. In addition, arabidopsis trichomes may serve as mechanosensors, possibly leading to specialized metabolite production when triggered [118,119]. *AtGL3*, a late wound-induced gene that shows higher stress-associated expression in WT compared with *lox2* plants (Pattern 5, Figure 5E), encodes a bHLH transcription factor that regulates trichome initiation as well as anthocyanin biosynthesis [120,121]. Constitutively, this transcription factor interacts with JAZ proteins, specifically, JAZ1, JAZ2, JAZ8 and JAZ11, leading to its repression [122]. In response to wounding, the resultant degradation of JAZ proteins coupled to increased *AtGL3* expression supports observations of a wound-associated increase in arabidopsis foliar trichomes [123].

#### 3.2.4. Chemical Defense

GSLs and flavonoids are important defensive compounds in the Brassicaceae [18,124,125]. A wound-induced difference in foliar GSL levels was not observed (Figure 7). However, in both genotypes, a strong expression of GSL biosynthetic genes was observed late after wounding, which corresponds to the light phase in our experiment. This supports previous observations of a possible role of circadian rhythms in foliar GSL biosynthesis [21].

In contrast, a genotype difference in wound-induced genes associated with flavonoid biosynthesis was identified in this study, with genes expressed more highly in WT plants (Pattern 5, Figure 6). Flavonoids can act as feeding or oviposition deterrents or toxins against insect herbivores [124,125]. Even though *AtRNS1*, a jasmonate-independent, wound-induced gene that encodes a ribonuclease that is a negative regulator of anthocyanin biosynthesis [126], is expressed in both genotypes (Pattern 4, Figure 5D), the late wound-induced expression of genes that encode transcriptional regulators or flavonoid biosynthetic enzymes was observed in WT plants (Pattern 5, Figure 5E). Thus, particularly in WT plants, there is a strong upregulation of genes involved in flavonoid biosynthesis, particularly anthocyanins, that may act as antioxidants or be involved in plant resistance against insect herbivory [125,127].

#### 3.2.5. Indirect Chemical Defense

Wounding of arabidopsis leaves results in the biosynthesis and release of volatile organic compounds (VOCs) that are involved in numerous ecological roles, including intra-plant stress signaling and tritrophic interactions, such as attracting parasitoids or predators of the herbivore [128,129]. From the diterpenoid precursor geranylgeranyl diphosphate, AtTPS04, which is expressed in arabidopsis non-glandular trichomes [50], catalyzes the production of (Ε,Ε)-geranyllinalool, which is converted by AtCYP82G1 into volatile 4,8,12-trimethyltrideca-1,3,7,11-tetraene (TMTT) [49], an important attractant in tritrophic interactions for beneficial parasitoid and predatory arthropods [130]. *AtTPS04* is induced in response to herbivory by caterpillars of the specialist diamond backmoth, *Plutella xylostella*, or the generalist African cotton leafworm, *Spodoptera littoralis* [131,132,133,134]. In our study, both *AtTPS04* and *AtCYP82G1* show early wound-induced gene expression (Pattern 1, Figure 5A). *AtCYP82G1* is also induced in response to the treatment of arabidopsis leaves with spider mite egg extract [113].

The expression of *AtTPS03* and *AtCYP81D11* is predominant in wounded WT plants (Pattern 2, Figure 5B). AtTPS03 encodes a terpene synthase that produces (*E*,*E*)-α-farnesene, an important component in highly attractive volatile blends attractive to parasitic wasps such as *Microplitis croceipes*, *Apanteles taragamae*, *Anaphes iole* and *Gonatocerus ashmeadi*, as well as acting as a deterrent to soybean cyst nematodes [135,136,137,138,139]. AtCYP81D11 also contributes to the volatile profile responsible for the attraction of parasitoid wasps, such as *Cotesia plutellae*, to wounded plants [140].

*AtHPL* had higher late wound-induced expression in WT compared with *lox2* plants (Pattern 5, Figure 5E). This jasmonate-responsive gene is also induced in response to herbivory by *P. rapae* or *S. littoralis* caterpillars [141]. Cytosolic AtHPL competes with the jasmonate biosynthetic pathway for 13-hydroxyperoxide precursors to generate alkenals, which are converted into C6 volatiles [83]. The AtHPL in the arabidopsis Col-0 ecotype (which was also used in this study) has a deletion resulting in a truncated protein that is unable to use 13-hydroperoxide linolenic acid as a precursor but uses 13-hydroperoxide linoleic acid to produce hexenals [142].

*AtBSMT1* was also more highly wound-induced in WT plants (Pattern 2, Figure 5B). This methyltransferase, involved in volatile methyl salicylate biosynthesis, is induced in response to *P. rapae* and *Pieris brassicae* herbivory [143,144]. The resultant methyl salicylate is an attractant for female parasitic *Diadegma semiclausum* wasps and also deters oviposition by female *P. brassicae* butterflies, thus decreasing herbivore damage [144,145].

In addition to the role that these VOCs play in attracting natural enemies of the herbivore [146], these volatiles can also act on the plant itself, leading to systemic upregulation of plant defense responses [147,148]. Recently, the importance of these volatiles as conspecific signals has been recognized [149]. Thus, volatiles may serve as systemic signals resulting in the induction of plant defense responses.

#### 3.2.6. Interference with Insect Nutrition or Physiology

Obtaining sufficient nitrogen to maintain development and fitness is a key challenge for phytophagous insects [150]. Thus, plants have numerous strategies to interfere with a herbivore’s ability to obtain sufficient nitrogen to limit herbivore success.

In herbivorous insects, serine proteinases, such as trypsin and chymotrypsin, initiate protein digestion [151]; However, plants produce inhibitors of these enzymes, known as proteinase inhibitors [152]. Overexpression of a soybean trypsin inhibitor in arabidopsis negatively affected the larval biomass of corn earworm, *Helicoverpa zea*, caterpillars [153]. In our study, we identified the expression of three wound-induced trypsin inhibitors. *AtTI1* is induced early in both genotypes and has previously been found to be induced by aphid attack (Pattern 1, Figure 5A) [154]. *AtKTI3* and *AtTI6* are more highly expressed in WT plants late after wounding (Pattern 5, Figure 5E).

Another late wound-induced gene identified in our transcriptomic study that interferes with nitrogen resources for the insect encodes arginase (AtARGAH2) (Pattern 5, Figure 5E). This enzyme catabolizes nitrogen-rich arginine to produce urea and ornithine. Direct feeding of *Manduca sexta* caterpillars on tomato plants overexpressing arginase resulted in smaller insects, presumably because of the decreased availability of the essential amino acid arginine [86]. However, this may also reflect the potential toxicity of arginase products, where ornithine may be converted by AtNATA1, a late pattern 4 gene (Figure 5D), to N^d^-acetylornithine [155]. This derivative negatively affects the fecundity of the green peach aphid, *Myzus persicae*. It is of interest that the pH optimum of plant arginase is alkaline [86], which likely allows it to be most active in its native cellular location, the mitochondrial matrix [156], and also in the alkaline midgut of caterpillars [157]. In addition to these activities, ARGAH2 activity may affect nitric oxide (NO) production [158]. Though this has been shown in the marine green algae *Ostreococcus tauri* [159], in higher plants, NOS-like activity is controversial; however, NO has been proposed to be released from arginine upon its conversion to citrulline [160,161]. Consistent with this, AtARGAH2 knockout plants had increased NO accumulation [158]. Differences in NO, or its more biologically stable form *S*-nitrosoglutathione (GSNO), can impact protein *S*-nitrosation and *S*-glutathionylation status of regulatory proteins involved in plant defense [162,163,164,165]. *Manduca sexta* caterpillars grew larger on plants silenced in their ability to produce GSNO. Methyl jasmonate-induced levels of proteinase inhibitors and some defensive specialized metabolites (caffeoylputrescine, diterpene glycosides) were lower in these plants [162]. Therefore, AtARGAH2 may affect caterpillar nutrition or plant defense.

The phloem-associated protein AtPP2-A5 has two domains, a PP2 (lectin activity) domain at the C-terminus and a Toll/Interleukin-1 receptor domain at the N-terminus [166]. Though this protein plays a major role in sealing wounded sieve elements (Pattern 1, Figure 5A), AtPP2-A5 overexpression or knockout lines show modified transcriptional patterns to spider mite herbivory, suggesting that the receptor portion of this protein recognizes an herbivore-specific effector to remodel gene expression [166]. The knockout line was more susceptible to spider mite damage, and the arthropods had higher mortality on the overexpression lines. This likely reflects direct interactions with the insect, potentially by binding to the arthropod gut epithelial [167]. Feeding aphids a diet spiked with recombinant AtPP2-A5 did not affect mortality but did negatively affect the weight gain of two different aphid species, the pea aphid *Acythosiphon pisum* (~30% smaller) and the green peach aphid *Myzus persicae* (10–20% smaller) [168]. Aphids reared on AtPP2-A5 overexpression lines had lower colonization and spent less time feeding on the phloem compared with WT plants [169].

Chlorophyllase, AtCHL1, catalyzes the hydrolysis of chlorophyll to chlorophyllide. Under stress conditions, this enzyme may be involved in chlorophyll degradation to minimize chlorophyll-associated ROS generation [170,171]. However, as this protein is associated with the endoplasmic reticulum or tonoplast, only upon cell disruption, such as that incurred by herbivory, does the enzyme come into contact with its substrate, chlorophyll. The product of this reaction, chlorophyllide, is toxic to *S. littoralis* caterpillars and binds to the midgut of *Bombyx mori* caterpillars, potentially impairing digestion [172]. However, other studies suggest that chlorophyllide may have roles that benefit the insect herbivore [173]. In our study, *AtChl1* was induced late after wounding and showed higher expression in WT plants (Pattern 5, Figure 5E).

## 4. Materials and Methods

### 4.1. Plant Maintenance

*Arabidopsis thaliana* wildtype (WT) Columbia (Col-0) seeds were obtained from the Arabidopsis Biological Resource Center. Seeds of the *lox2-1* mutant line, which has a mutation in the tryptophan amino acid at position 630 to produce a stop codon resulting in a truncated non-functional protein, were generously provided by Dr. E. E. Farmer [28]. The seeds were surface-sterilized in 70% (*v*/*v*) ethanol for one min, then 0.6% (*v*/*v*) bleach (NaOCl) for 3 min, followed by five successive washes in sterile ddH_2_O. Between these treatments, the seeds were recovered by centrifugation, and the liquid was removed. The seeds were then placed in Petri dishes containing Murashige and Skoog salts, pH 5.8, in 0.8% agar, followed by stratification in the dark at 4 °C to promote synchronized germination. After 2 days, the Petri dishes were transferred to a growth cabinet with 14 h of light with an intensity of 250 μmol m^−2^ s^−1^ followed by 10 h darkness. The temperature of the light–dark cycle was 23:20 °C. After one week, the germinated seedlings were transplanted into pots (12 cm diameter × 11 cm height) containing Fafard Agromix G6 potting medium and grown under the same conditions. Plants were bottom-watered 3 times per week with 20:20:20 NPK fertilizer (0.14 g/L distilled water) and used for experimentation at the vegetative 3.9 stage [174].

### 4.2. Experimental Design

Two days prior to the wounding experiment, a plexiglass sheet was placed between randomly chosen plants that were to remain unwounded or mechanically damaged to separate the treatments and avoid volatile signaling between these groups. At 9 PM (time 0; dark phase), arabidopsis lines were either mechanically damaged, whereby each leaf of the rosette was wounded once by a hole punch, without harming the mid-vein, or left unwounded. Whole rosettes were collected for phytohormones (1 h; dark phase), GSLs (12 h; light phase) or transcriptomics (1 h and 12 h, dark and light phase, respectively) post-damage, flash-frozen in liquid nitrogen and stored at −80 °C until analysis. The experiment was repeated temporally three times to collect samples for transcriptomics and five times for phytohormone and GSL analyses. At each temporal replicate, one sample was taken for the different analyses (RNA-Seq or metabolite analysis) of transcriptomics (n = 3), phytohormones (n = 5) and GSLs (n = 5).

### 4.3. Phytohormone Analysis

Following the protocol described in Martinez Henao et al. [175], plant samples were finely ground and extracted in ethyl acetate containing isotopically labeled standards (D6-JA, D6-JA-Ile and D4-SA (OlChemim, s.r.o)). The samples were vigorously vortexed and centrifuged (19,000× *g*, 10 min, 4 °C), and the supernatant was transferred to a new tube. The extraction was repeated, and the supernatants were pooled. Following evaporation using a vacuum concentrator at room temperature, the resulting pellet was resuspended in 70% (*v*/*v*) methanol (HPLC-MS grade). A final centrifugation step was performed as above to ensure the removal of all non-soluble debris. Metabolites were separated by ultrahigh performance liquid chromatography (UHPLC) followed by detection on a triple quadrupole mass spectrometer (EVOQ-TQ-MS, Bruker, Hamburg, Germany). Reverse phase UHPLC was performed using a Zorbax Extend-C18 column (4.6 × 50 mm, 1.8 μm, Agilent Technologies, Santa Clara, CA, USA). The mobile phase began with 5% (*v*/*v*) acetonitrile (ACN), 0.05% (*v*/*v*) formic acid for 30 s and then increased to 50% (*v*/*v*) ACN, 0.05% (*v*/*v*) formic acid over 2 min. After separation, the compounds were nebulized by electron spray ionization and detected using the EVOQ-TQ-MS. Phytohormones were identified based on their retention time, in comparison with known standards, as well as their *m*/*z*.

### 4.4. Glucosinolate Analysis

GSLs were extracted from lyophilized leaf tissue and analyzed by high-performance liquid chromatography (HPLC)-pulsed amperometric detection following Grosser and van Dam [176]. Briefly, 70% MeOH was added to the finely ground tissues and incubated at 90 °C for 6 min to inactivate myrosinases followed by sonication for 15 min. After centrifugation (2975× *g* for 10 min), the supernatant was transferred to a clean tube, and the pellet was re-extracted. Pooled supernatants were passed through a diethylaminoethyl Sephadex A-25 ion exchange column preconditioned with ddH_2_O. After washing (2 × 1 mL 70% MeOH, 2 × 1 mL ddH_2_O, 1 × 1 mL 20 mM sodium acetate buffer, pH 5.5), the column was treated with 10 U of arylsulfatase and incubated for 12 h at RT. Desulfated GSLs were eluted in sterile MilliQ H_2_O (2 × 0.75 mL) and lyophilized.

GSLs were separated by reverse-phase chromatography on a C_18_ column (Alltima C_18_, 150 × 4.6 mm, 3 μm, Alltech, Lexington, USA) using a mobile gradient from 2% acetonitrile (ACN) to 35% ACN in 30 min at a flow rate of 0.75 mL min^−1^. GSLs were identified based on retention time to known standards (glucoiberin (3-methylsulfenylpropyl GSL), glucoerucin (4-methylthiobutyl GSL), progoitrin (2-hydroxy-3-butenyl GSL), sinigrin (2-propenyl GSL), gluconapin (3-butenyl GSL), glucobrassicanapin (4-pentenyl GSL), glucobrassicin (indol-3-ylmethyl GSL), sinalbin (4-hydoxybenzyl GSL), glucotropaeolin (benzyl GSL) and gluconasturtiin (2-phenylethyl GSL); Phytoplan, Heidelberg, Germany)) and UV spectra. GSL concentrations were calculated from a sinigrin standard curve according to Grosser and van Dam [176].

### 4.5. RNA Extraction, Library Preparation and Transcriptomics

Total RNA was extracted from flash-frozen pulverized plant rosettes (~100 mg) using the RNeasy Plant Mini Kit (Qiagen, Venlo, The Netherlands) following the manufacturer’s protocol with an additional on-column DNase digestion step to avoid genomic DNA contamination. The 2100 Bioanalyzer instrument (Agilent Technologies) was used to determine RNA quality using an RNA integrity number (RIN) of 8.7 as a minimum threshold. Total RNA samples were processed by Genome Québec Innovation Centre for library preparation and next-generation RNA sequencing (RNA-Seq). Libraries were generated from 250 ng of total RNA as follows: mRNA enrichment and ribosomal RNA removal were performed using the Next Poly(A) Magnetic Isolation following the manufacturer’s instructions (New England BioLabs, Ipswich, USA). cDNA synthesis was performed with Next RNA First Strand Synthesis and Next Ultra Directional RNA Second Strand Synthesis kits (New England BioLabs). The remaining steps of library preparation were performed using the Next Ultra II DNA Library Prep Kit for Illumina (New England BioLabs). Adapters and PCR primers were purchased from New England BioLabs.

The libraries were normalized, pooled and denatured in 0.02 N NaOH, followed by neutralization using HT1 hybridization buffer. Twenty-four strand-specific mRNA libraries were generated and sequenced by loading 200 pM on the NovaSeq 6000 S4 (Illumina) using the Xp protocol as per the manufacturer’s recommendations, with paired-end (PE) mode for 2 × 100 base pairs (bp) resulting in >25 million reads in each direction. The Illumina phiX control v3 library was used as a control and mixed with libraries at a 1% level. Base calling was performed using Real-Time Analysis (RTA) v3 software. The program bcl2fastq2 v2.20 was used to demultiplex samples and generate fastq reads.

### 4.6. Quality Check, Clipping and Mapping

Raw read quality was examined by performing FastQC 0.11.9 on “.fastq.gz” file [177]. FastQC produces a control statistic and evaluates every metric using a classification system of pass, warn or fail. The adapter clipping and bad end quality trimming were performed by subjecting the raw reads to fastp tool version 0.20.1 to remove adapters, low-quality bases with a Phred score less than 20 and reads shorter than 25 bp from the tail (3’ end) from further analysis [178]. This preprocessing was followed by another quality check with FastQC. Read mapping was conducted using a splice-aware alignment tool STAR v2.7.9a to align the trimmed reads against the arabidopsis reference genome downloaded from The Arabidopsis Information Resource (TAIR, assembly ID: TAIR10, https://www.arabidopsis.org/download_files/Genes/TAIR10_genome_release/TAIR10_gff3/TAIR10_GFF3_genes.gff (accessed on 9 December 2022)) [179].

### 4.7. Differential Gene Expression

Strand-specific transcript abundance was calculated using featureCounts (a tool included in the subread package 2.0.3) on mapped and sorted BAM files. The resulting read counts per transcript values were imported into the web-based tool NetworkAnalyst 3.0 for data filtering, Log_2_ normalization and differential analysis [180]. The statistical method DESeq2 with a negative binomial (Gamma–Poisson) distribution was used to identify differentially expressed genes using the log_2_-fold change (logFC) calculation [181,182]. Genes with a False Discovery Rate (FDR)-corrected *p*-value (padj) ≤ 0.05 and log_2_ fold change ≥ 2 or ≤−2 were deemed significant. The transcriptomic data generated by this study are available in the Supplementary Materials of this article (Supplemental Table S1) and the raw read data (FASTQ) were deposited in the NCBI Sequence Read Archive (Bioproject ID #PRJNA1077722).

Transcriptomic visualization (ridgeplots, heatmaps, principal component analysis (PCA)) was performed using MetaboAnalyst 5.0 and ExpressAnalyst [183,184]. For visualization, the data were normalized by log_10_ transformation.

### 4.8. Statistics

For phytohormone and GSL analyses, a two-factor analysis of variance (ANOVA) (factors: genotype and treatment) was conducted using the statistical program SPSS vers. 29 (Supplemental Tables S2 and S3).

## 5. Conclusions

By comparing early (1 h) and late (12 h) constitutive and wound responses between arabidopsis WT and *lox2* mutants, we identified gene expression differences related to the regulation of jasmonate biosynthesis and, in mechanically damaged plants, resistance to insect herbivores. Both constitutively and in wounded plants, SA levels are higher in *lox2*, which is reflected in the expression of SA-responsive genes. Since jasmonate and SA pathways are mutually antagonistic [106], this suggests that AtLOX2 may be involved in the constitutive biosynthesis of jasmonates that modulate SA levels.

**Figure 8:**
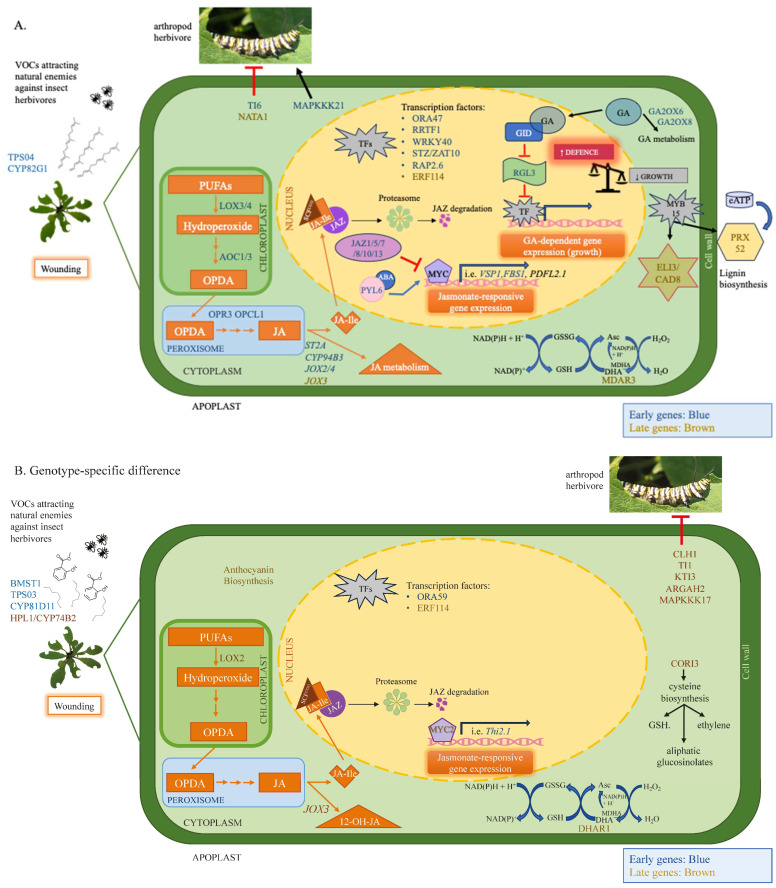
Wound-induced genes involved in plant–insect interactions in arabidopsis WT and *lox2* mutants. Foliar gene expression in wounded *lox2* mutants, which has a truncated non-functional enzyme, was compared to WT plants. Proteins encoded by early genes (1 h) are shown in blue, whereas later transcript expression (12 h) is depicted in brown. (**A**)**.** General wound-induced responses found in both genotypes. Jasmonate biosynthesis begins in the chloroplast from 18C membrane lipid-derived precursors, typically α-linolenic acid, to finally form one of the biologically active jasmonates, jasmonoyl-isoleucine (JA-Ile). Numerous genes encoding enzymes in the jasmonate biosynthetic pathway are upregulated in wounded plants. JA-Ile enters the nucleus and forms a bridge between the SCF^COI1^ and jasmonate ZIM domain (JAZ) repressors, leading to their degradation through the proteasome. The degradation of JAZ proteins releases MYC2/3/4 transcription factors, leading to jasmonate-responsive gene expression. The abscisic acid (ABA)-PYL6 receptor complex positively interacts with MYC2. Genes encoding JAZ-negative regulators as well as those further metabolizing jasmonic acid (JA) (i.e., JOX, ST2A) to inactive derivatives are also wound-induced. Gibberellin (GA) bound to its receptor GID1 activates a pathway that leads to the proteasome-mediated degradation of negative DELLA growth regulators, such as RGL3. In wounded leaves, the increase in *RGL3* expression and genes that encode GA2OX6 and GA2OX8, involved in gibberellin metabolism to inactive products, results in the suppression of plant growth. Numerous genes involved in oxidative stress are wound-induced. For example, the oxidative stress-associated transcription factor RRTF1 and MDAR3, which are part of the Foyer–Halliwell–Asada cycle, a series of interconnected enzymatic reactions to detoxify the reactive oxygen species hydrogen peroxide (H_2_O_2_). Wound-induced MYB15 leads to the expression of *ELI3/CAD8* and *PRX52*, which contribute to lignin biosynthesis. TPS04 and CYP82G1 are involved in the biosynthesis of volatiles, such as 4,8,12-trimethyltrideca-1,3,7,11-tetraene (TMTT), that are attractive to natural enemies of the herbivorous insect. TI1 and NATA1 contribute to plant resistance against arthropods, whereas MAPKKK21 is a negative regulator of arthropod resistance. (**B**)**.** LOX2-specific responses. Wound-induced genes expressed at higher levels in WT compared with lox2 plants. Wound-induced expression of genes that encode LOX2 and MYC2 involved in jasmonate biosynthesis and signaling, respectively, is noted. CORI3 is a cysteine lyase involved in cysteine biosynthesis that produces precursors for ethylene, aliphatic glucosinolates (GSLs) and reduced glutathione (GSH) involved in the Foyer–Halliwell–Asada cycle. Also, the expression of the gene encoding DHAR1 in this pathway is wound-induced. Other cellular antioxidants whose biosynthetic pathway is positively regulated in these plants are anthocyanins. Indirect defenses involved in volatile biosynthesis are wound-induced. An increase the genes that encode proteins involved in antinutritive defenses, such as CLH1, ARGAH2 and TIs, as well as MAPKKK17, an important signaling kinase involved in arthropod resistance, is also seen. Abbreviations: ABA: abscisic acid, AOC: allene oxide cyclase, AOS: allene oxide synthase, DHAR1: dehydroascorbate reductase1, GA: gibberellin, GSH: reduced glutathione, GSL: glucosinolate, GSSG: oxidized glutathione, JA: jasmonic acid, JA-Ile: jasmonoyl-isoleucine, JAZ: jasmonate-Zim domain, JOX: jasmonate oxidase, LOX: lipoxygenase, OPDA: 12-*oxo*-phytodienoic acid, OPR: *oxo*-phytodienoate reductase, OPCL: OPC-8-CoA ligase, PUFA: polyunsaturated fatty acid, TF: transcription factor, TMTT: 4,8,12-trimethyltrideca-1,3,7,11-tetraene.

As expected, the wound-associated jasmonate burst is dampened in *lox2* compared with WT plants. In the early response to wounding, genes are similarly expressed in both genotypes with few genotype-specific differences. In contrast, in the later transcriptional responses, higher expression of numerous genes involved in insect resistance is observed in WT plants compared to *lox2*, highlighting the role of AtLOX2 in arabidopsis resistance to insects (Figure 8).

## Data Availability

The data that support the findings of this study are available within this article. The transcriptomic data (FASTQ) generated by this study are available through the NCBI Sequence Read Archive (Bioproject ID #PRJNA1077722).

## Supplementary Materials

The following supporting information can be downloaded at: https://www.mdpi.com/article/10.3390/ijms25115898/s1.

## Abbreviations

ABA: abscisic acid, ACN: acetonitrile, ANOVA: analysis of variance, AOC: allene oxide cyclase, AOS: allene oxide synthase, BPs: base pairs, Col-0: Columbia, DEGs: differentially expressed genes, EDS1: enhanced disease susceptibility 1, GSH: reduced glutathione, GSSG: oxidized glutathione, JA-Ile: (+)-7-*iso*-jasmonoyl-isoleucine, JAR1: jasmonate resistant1, JAZ: jasmonate zim domain, KOBAS: KEGG orthology-based annotation system, LOX: lipoxygenase, MACP: membrane attack complex/perforin, OPDA: (9*S*,13*S*)-12-*oxo*-phytodienoic acid, PCA: principal component analysis, PE: paired-end, PR1: pathogenesis-related 1, RIN: RNA integrity number, RNA-Seq: RNA sequencing, ROS: reactive oxygen species, TIR-NBS-LRR: toll interleukin receptor–nucleotide binding site–leucine-rich repeat, UHPLC-EVOQ-TQ-MS: ultrahigh performance liquid chromatography–Evoq–triple quadrupole–mass spectrometry, VOCs: volatile organic compounds, VSP: vegetative storage protein.
