# Supplementary material for: Arabidopsis Transcriptomics Reveals the Role of Lipoxygenase2 (AtLOX2) in Wound-Induced Responses
Source: Int J Mol Sci. 2024 May 28;25(11):5898. doi: 10.3390/ijms25115898 (PMC11173247; doi:10.3390/ijms25115898)

Supplemental Figures

Supplemental Fig. S1. Foliar abscisic acid (ABA) levels. Four week-old *Arabidopsis thaliana* wildtype or *lox2* plants were left undamaged (U, ■) or wounded (W, ▩) with a hole punch on each fully expanded rosette leaf and harvested 1 h post-damage. ABA levels are represented by the mean ± SE. Differences in phytohormone levels were determined by two-factor analysis-of-variance (2-factor ANOVA)(Factors: genotype, treatment) followed by Tukey HSD (**Supplemental Table S2**).

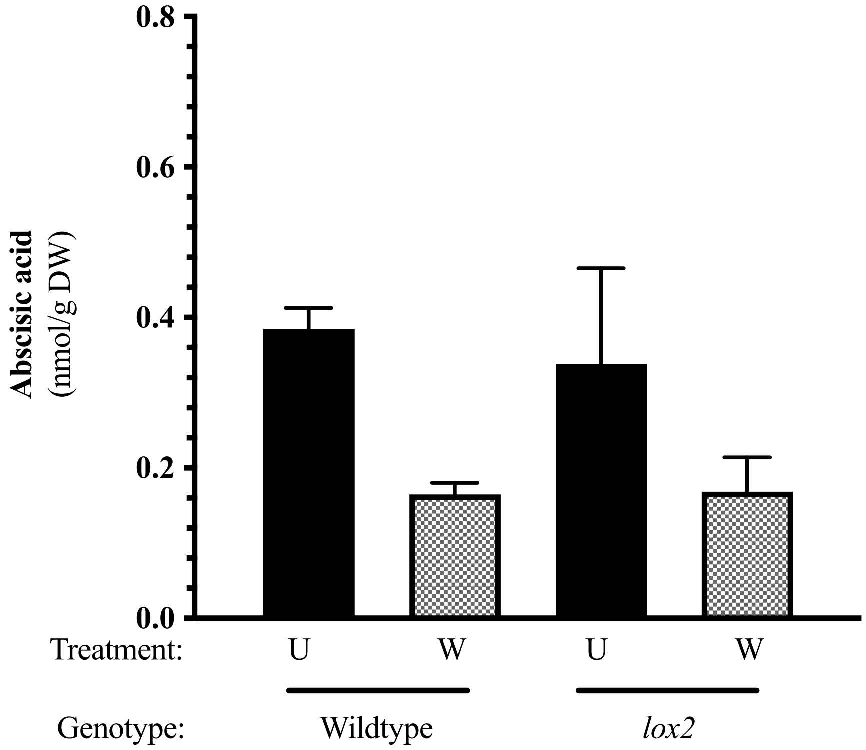

Supplemental Fig. S2. Singlet ($^1O_2$) oxygen-responsive gene expression. AtLOX2 has been implicated in $^1O_2$ production [34,35]. Four week-old *Arabidopsis thaliana* wildtype (WT) or *lox2* plants were undamaged (U) or wounded (W) with a hole punch on each fully expanded rosette leaf and harvested at 1 h. $^1O_2$-responsive genes were visualized by a heatmap [84].

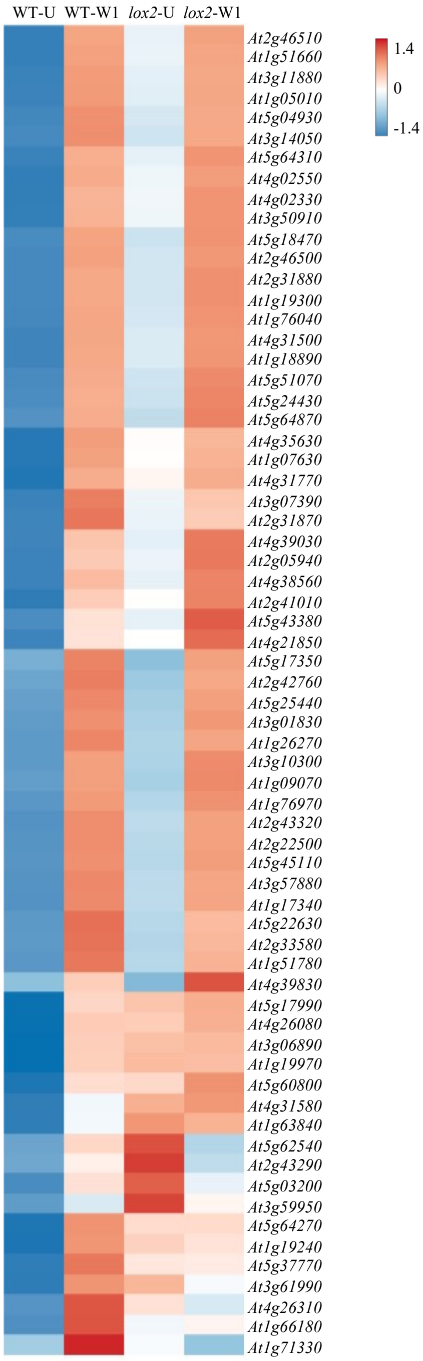

Supplement: Supplementary file 1 [file ijms-25-05898-s001.zip › 18 KAUR Supplemental Fig 2024.pdf]
